# Electron crystallography

**DOI:** 10.1107/S2052252519011497

**Published:** 2019-08-29

**Authors:** Alexander J. Blake, Marc de Boissieu, Ashwini Nangia

**Affiliations:** a School of Chemistry, The University of Nottingham, University Park, Nottingham, NG7 2RD, United Kingdom; b Univ. Grenoble Alpes, CNRS, SIMaP, F-38000 Grenoble, France; c Chemistry, CSIR-National Chemical Laboratory, Dr. Homi Bhabha Road, Pune, MH 411008, India

**Keywords:** electron crystallography, electron diffraction, data processing

## Abstract

A Special Issue on the topic of Electron Crystallography, now available in the August 2019 issue of *Acta Crystallographica, Section B*, contains contributions which we hope will interest readers of **IUCrJ**.

Since its launch in 2014, **IUCrJ** has published 111 articles with the term ‘electron crystallography’ as one of the keywords, with these articles attracting 450 citations a year and comparable activity continuing into 2019. This level of relevant activity provides the context and justification for the more specialized, in-depth, Special Issue on Electron Crystallography now available in the August 2019 issue of *Acta Crystallographica, Section B*, which contains important contributions from many of the major players in the field. We therefore believe that this Special Issue will prove attractive and relevant to the readership of *IUCrJ* and we wish to recommend the articles therein, as described in the Guest Editors’ Introduction reproduced below.

**Introduction by the Guest Editors of the Special Issue on Electron Crystallography** (Joke Hadermann and Lukáš Palatinus)

Structure analysis of micro- and nanocrystalline materials has witnessed immense progress in the last decade thanks to the development of electron diffraction techniques. The automation of data collection, development of new data collection modes and improvements in the data treatment have allowed unprecedented progress in most aspects of crystallography dealing with very small crystals. Probably the most notable change of paradigm is observable in the structure determination of unknown phases by electron diffraction. Three-dimensional diffraction techniques now allow almost routine solution and refinement of structures from single crystals as small as a few tens of nanometres, providing access to hitherto unsolvable crystal structures or to previously unattainable levels of structural detail. Scanning diffraction techniques allow phase and orientation mapping with nanometre resolution and even three-dimensional reconstruction of phase and orientation distributions.

This special issue features a collection of original contributions covering a broad range of aspects of electron crystallography. An interested reader will find papers describing the foundations and methodological basis of structure solution by electron diffraction (Eggeman, 2019 ▸; Kolb *et al.*, 2019 ▸; Gemmi & Lanza, 2019 ▸), theoretical and methodological advances in data processing (Rauch & Véron, 2019 ▸; Palatinus *et al.*, 2019 ▸; Latychevskaia & Abrahams, 2019 ▸), discussion of applications of electron diffraction outside the realm of perfectly periodic crystals (Gorelik *et al.*, 2019 ▸; Mugnaioli & Gorelik, 2019 ▸) as well as specific case studies showing the application of the methods to hot topics in current crystallography (Wang *et al.*, 2019 ▸; Hadermann & Abakumov, 2019 ▸).

The collection of contributions in this special issue showcases the diversity of applications of current electron diffraction techniques, demonstrates the state of the development of the technique and also features work that further advances the electron diffraction methods. We believe that this special issue can serve as a starting point for anybody interested in electron crystallography and we are convinced that the contributions in this issue will become reference points for future research in this exciting field.
